# The success rate of robotic natural orifice intracorporeal anastomosis and transrectal extraction (NICE procedure) in a large cohort of consecutive unselected patients

**DOI:** 10.1007/s00464-022-09717-6

**Published:** 2022-11-23

**Authors:** Eric M. Haas, Thais Reif de Paula, Roberto Luna-Saracho, Melissa S. Smith, Jose I. Ortiz De Elguea-Lizarraga, Roberto Secchi del Rio, Mark Edgcomb, Jean-Paul LeFave

**Affiliations:** 1 University of Houston College of Medicine, Chief Quality Colon and Rectal Surgery, HCA Healthcare Gulf Coast Division, 6560 Fannin Street, Houston, TX 77030 USA; 2 Houston Colon Foundation, Houston, TX USA

**Keywords:** NICE procedure, Intracorporeal anastomosis (ICA), Natural orifice transrectal extraction, Robotic surgery, Colorectal surgery, Total intracorporeal surgery

## Abstract

**Background:**

The Robotic NICE procedure is a total intracorporeal natural orifice approach in which specimen extraction and anastomosis is accomplished without an abdominal wall incision other than the port sites themselves. We aim to present the success rate of the NICE procedure in a large cohort of unselected consecutive patients presenting with colorectal disease using a stepwise and reproducible robotic approach.

**Methods:**

Consecutive patients who presented with benign or malignant disease requiring left-sided colorectal resection and anastomosis between May 2018 and June 2021 were evaluated. Data abstracted included demographic, clinical data, disease features, intervention data, and outcomes data. The main outcome was success rate of Intracorporeal anastomosis (ICA), transrectal extraction of specimen (TRSE), and conversion rate.

**Results:**

A total of 306 patients underwent NICE procedure. Diverticulitis was the main diagnosis (64%) followed by colorectal neoplasm (27%). Median operative time was 219 min, and the median estimated blood loss was 50 ml. ICA was achieved in all cases (100%). TRSE was successfully achieved in 95.4% of cases. In 14 patients (4.6%), an abdominal incision was required due to inability to extract a bulky specimen through the rectum. There overall postoperative complications rate was 12.4%. Eight patients (2.6%) experienced postoperative ileus. There were no superficial or deep surgical site infection (SSI). Eleven patients (3.6%) developed organ SSI space including 5 patients with intra-abdominal abscess and 4 patients with anastomotic leak. There was one mortality (0.3%) due to toxic megacolon from resistant Clostridium difficile. The 30-day reoperation rate was 2.9% (*n* = 9) including six patients presenting with organ space SSI and three patients with postoperative obstruction at the diverting loop ileostomy site.

**Conclusion:**

The NICE procedure is associated with a very high success rate for both intracorporeal anastomosis and transrectal specimen extraction in a large cohort of unselected patients.

Advancements in da Vinci (Intuitive Surgical, Sunnyvale, CA, USA) robotic technology have resulted in a surge of interest in intracorporeal and natural orifice approaches in colorectal surgery. The feasibility and merits of intracorporeal anastomosis (ICA) for right-sided ileocolic resections have been explored in earnest over the last 10 years. Patient benefits such as faster return of bowel function, lower rates of prolonged postoperative ileus and lower rates of incisional hernia have been shown in randomized controlled trials, comparative studies and matched studies [1–4].

For left-sides cases involving colorectal anastomosis, it is theorized, that in addition to the merits of ICA, natural orifice transrectal specimen extraction (TRSE) carries even more benefits by avoiding an abdominal wall incision. This approach was pioneered in laparoscopic surgery by Franklin et all in 1993 [5], and several studies have since shown promising patient benefits including less pain, lower opioid use and fewer wound complications [6–10]. Yet significant technical challenges has greatly hindered widespread adaption.

In 2018, we reported on the successful utilization of the Robotic Xi technology to facilitate left-sided colorectal resection with transrectal extraction of specimen and intracorporeal anastomosis which we termed the Robotic NICE procedure [11, 12]. The robotic platform helps overcome many of the challenges of this total intracorporeal approach and we have since optimized this technique and developed a reproducible stepwise procedure [13]. We present our series of NICE cases for both benign and malignant disease over a 3-year period. We hypothesize that the Robotic NICE procedure can be performed successfully in consecutive and unselected cohort of patients undergoing elective left-sided colorectal resections.

## Methods

### Patients

Consecutive patients who presented with benign or malignant disease requiring left-sided colorectal resection and anastomosis between May 2018 and June 2021 were evaluated. All patients underwent Robotic Natural orifice Intracorporeal anastomosis with transrectal Extraction of specimen (NICE Procedure) consisting of either Anterior Resection (AR) or Low Anterior Resection (LAR). Patients who received neoadjuvant radiation with planned diverting loop ileostomy were excluded. All the cases were performed by a single surgeon (EMH) and the colorectal surgery team in the Texas Medical Center. All patients underwent preoperative workup with physical examination, colonoscopy, imaging, and laboratory exams as required. All patients were recovered on a standardized enhanced recovery program (ERP) including multimodal post-operative pain control.

### Surgical technique

For benign cases three 8 mm robotic ports and a 5 mm Air Seal port (Optiview, Inc., Jacksonville, FL, USA) are utilized. Once placed, the patient is positioned with 8-degree left side elevation and 18–20 degrees of Trendelenburg and the robot is docked. A lateral to medial mesenteric sparing approach is performed [11, 13]. The dissection commences by incising the white line of Toldt along the left colon and exposing the paracolic gutter by retracting on the colonic mesentery. Splenic flexure mobilization is then performed when indicated for a tension free anastomosis. Attention is then drawn to the pelvis and the lateral attachments along the intersigmoid fold are released. The right and left rectal reflection is then incised as is the anterior reflection to release the peritoneal attachments of the rectum. The proximal level of resection is then identified and divided using the vessel sealer extend (VSE) with minimal use of energy. A mesenteric sparing dissection is performed staying close to the bowel, preserving the superior rectal artery and avoiding the retroperitoneal plane. Dissection is carried to the distal level of resection along the rectum which is then transected with the VSE. Sizers are then passed through the rectal cuff followed by placement of the small Alexis wound retractor (Applied Medical, Rancho Santa Margarita, CA, USA). Transrectal extraction of the specimen is then performed. In benign cases in which there is a bulky specimen, a thinning maneuver is performed in which the mesentery is detached from the surface of the bowel wall to facilitate transrectal extraction. The retractor is then removed in preparation for the end-to-end anastomosis.

For malignant disease, the port site configuration is the same as benign disease except a 12 mm robotic port (instead of an 8 mm port) is placed in the right lower quadrant to facilitate the use of a robotic stapler. On occasion an additional 8 mm port is placed in the left lower quadrant for deep dissection in a narrow pelvis. The linear stapler (SureFormTM60) is used to divide and close the specimen to avoid intraabdominal contamination in the presence of malignant pathology. A medial to lateral approach is performed with high ligation of the pedicle and oncologic lymph node dissection which has been previously described [12]. Following the dissection and mobilization, the robotic linear stapler is used to divide the bowel at the proximal and distal level of resection thereby keeping the specimen intact. The staple line along the proximal colon and rectum is then excised and removed. The small Alexis retractor is placed across the rectum and the specimen is extracted. The retractor is then removed in preparation for the end-to-end anastomosis.

In cases in which there is a concern for trauma to the rectum due to a bulky specimen, we complete the anastomosis and thereafter extract the specimen through a Pfannenstiel incision.

### Intracorporeal end-to-end anastomosis

The anvil to the circular stapler (CDH 29 mm or 31 mm circular stapler, Ethicon, Somerville, NJ, USA) is delivered through the rectum into the pelvis. A 6-inch 3.0 V-Loc suture on a v20 needle (V-Loc 180™, Covidien; Mansfield, MA, USA) is used to place a purse-string suture around the cut edge of the proximal bowel. The anvil is inserted, and the purse-string suture is tightened. Next a vicryl Endoloop® (Endoloop®, Ethicon, Somerville, NJ, USA) is placed about the neck of the tissue to further secure the anvil. Attention is then drawn to the divided edge of the rectum and a second V-Loc pursestring suture is placed. The spike of the circular stapler is then advanced and the pursestring suture is tightened about the spike. The anvil and the spike are seated, and an anastomosis is fashioned. In the majority of cases of malignant disease, the rectal cuff is closed using the linear stapler. When performed, a handsewn end-to-end anastomosis was achieved with the use of two or more 3.0 V-Loc suture on a v20 in a single layer. The integrity of the anastomosis is assessed with both direct endoscopic visualization as well as air insufflation test.

### Study variables and outcome measures

Demographic data including age, sex, and Body Mass Index (BMI) as well as clinical data, including American Society of Anesthesiology (ASA), previous abdominal surgery, and diagnosis were abstracted. Diverticulitis was classified as recurrent or complicated which consisted of the presence of abscess, fistula or stricture. The intervention data included type of surgery, operative time, estimated blood loss, intraoperative transfusion, splenic flexure takedown, site of specimen extraction, anastomosis type, anastomosis location, diverting loop ileostomy, intraoperative complications, and conversion to open or other minimally invasive approach. The surgical pathology data were abstracted for the subset of patients with malignant disease. The surgical outcomes analyzed were return of bowel function, length of hospital stay, 30-day postoperative complications, 30-day unplanned readmissions, 30-day unplanned reoperations, and 30-day mortality. The primary outcomes consisted of success rate of intracorporeal anastomosis (ICA), success rate of transrectal extraction of specimen (TRSE) and conversion rate.

### Data analysis

Summary statistics were presented for all data. Categorial variables were presented as count and percentages. Continuous variables were presented as median or mean, as appropriate, and range. This study was approved by the Houston Methodist Hospital Internal Review Board (study protocol 00012111).

## Results

There was a total of 306 patients who underwent Robotic NICE left-sided resection of which 54% were female. The median age was 59 years-old (range, 19–88) and the median BMI was 27.8 kg/m2 (range, 17.2–55.6) of which 34.3% (105 patients) presented with BMI ≥ 30 kg/m^2^. The majority of patients were ASA II (59.8%) and just over half (52.9%) had a previous abdominal surgery. Diverticulitis was the main diagnosis (64%) followed by colorectal neoplasm (27%) and other (9%). In the subgroup of patients with diverticulitis, 47.4% had complicated disease (abscess, fistula and/or stenosis). Demographic and clinical features are presented in Tables 1 and 2.

**Table 1:** Demographic and clinical features of patients undergoing Robotic NICE procedure

	All cases *n* = 306 (100%)	Diverticulitis *n* = 196 (64.1%)	Neoplasm *n* = 83 (27.1%)	Other *n* = 27 (8.8%)
Age in years, median (IQR)	59 (19–88)	59 (22–84)	61 (31–88)	39 (19–83)
Sex				
Female	166 (54.2)	111 (56.6)	32 (38.6)	23 (85.2)
Male	140 (45.8)	85 (43.4)	51 (61.4)	4 (14.8)
BMI kg/m^2^, median (IQR)	27.8 (17.2–55.6)	28.5 (18.3–55.6)	27.3 (18.2–45)	26.1 (17.2–38.4)
ASA				
II	183 (59.8)	120 (61.2)	42 (50.6)	21 (77.8)
III	120 (39.2)	75 (38.3)	39 (47)	6 (22.2)
IV	3 (1)	1 (0.5)	2 (2.4)	0 (0)
Previous abdominal surgery	162 (52.9)	111 (56.6)	32 (38.6)	19 (70.4)

All values are count (percentages), unless specified otherwise

*NICE* Natural orifice intracorporeal anastomosis with transrectal extraction, *BMI* Body Mass Index, *ASA* American society of anesthesiology

**Table 2:** Diagnosis

	*n* = 306 (100)
Recurrent diverticulitis	103 (33.6)
Complicated Diverticulitis	93 (30.4)
Abscess	36 (11.7)
Fistula	45 (14.8)
Stricture	12 (3.9)
Colorectal neoplasm	83 (27.1)
Other	
Endometriosis	15 (4.9)
Rectal prolapse	6 (2)
Crohn’s/colitis	6 (2)

All values are count (percentages), unless specified otherwise

In terms of the procedure type, the majority were anterior resection (54.2%) followed by low anterior resection (45.8%). The overall median operative time was 219 min (range, 99–510) and the median estimated blood loss was 50 ml (range, 0–600). The median operative time for those with neoplasm was 230 min, for complicated diverticulitis was 222 min, and for recurrent diverticulitis was 186 min. Splenic flexure mobilization was performed in 83.7% of the patients. Intraoperative outcomes are summarized in Table 3.

**Table 3:** Intraoperative variables of patients undergoing Robotic NICE procedure

	All cases *n* = 306 (100)	Diverticulitis *n* = 196 (64.1)	Neoplasm/cancer *n* = 83 (27.1)	Others *n* = 27 (8.8)
Da Vinci model Xi	306 (100)	196 (100)	83 (100)	27 (100)
Type of surgery				
AR	166 (54.2)	121 (61.7)	35 (42.2)	10 (37)
LAR	140 (45.8)	75 (38.3)	48 (57.8)	17 (63)
Operative time in minutes, median (range)	219 (99–510)	204.5 (99–449)	230 (118–510)	*203.5 (110–353)
Estimated blood loss in ml, median (range)	50 (0–600)	50 (0–300)	50 (10–200)	50 (25–600)
Intraoperative transfusions	0 (0)	0 (0)	0 (0)	0 (0)
Diverting loop ileostomy	40 (13.1)	27 (13.8)	11 (13.3)	2 (7.4)
Splenic flexure takedown	256 (83.7)	182 (92.9)	69 (83.1)	5 (18.5)
Intraoperative complications**	5 (1.6)	0 (0)	0 (0)	0 (0)

All values are count (percentages), unless specified otherwise

*NICE* Natural orifice intracorporeal anastomosis with transrectal extraction, *AR* Anterior resection, *LAR* Low anterior resection

*Operative time not reported in cases involving more than one specialty

**Three patients had rectal tear during extraction, two patients had a splenic capsular tear

Transrectal extraction of specimen was successfully achieved in 292 patients (95.4%). In 14 patients (4.5%), an abdominal incision was required due to inability to extract a bulky specimen through the rectum. Of these cases, 13 had malignant disease. Of the 223 patients with benign disease, 36.3% (*n* = 81) underwent a thinning maneuver to facilitate transrectal extraction. There was a total of 5 (1.6%) intraoperative complications. In three patients, trauma during the extraction process resulted in a rectal tear that required proctorrhaphy. In two patients a splenic capsular tear was encountered during splenic flexure takedown. In all cases (100%), an intracorporeal end-to-end colorectal anastomosis was successfully achieved either with a circular stapler (93.5%) or handsewn technique (6.5%). There were no conversions to open or other MIS approaches. Table 4.

**Table 4:** Success rate of TRSE and ICA

	All cases *n* = 306 (100)	Diverticulitis *n* = 196 (64.1)	Neoplasm/cancer *n* = 83 (27.1)	Others *n* = 27 (8.8)
Specimen extraction				
Transrectal	292 (95.4)	195 (99.5)	70 (84.3)	27 (100)
Transabdominal	14 (4.6)	1 (0.5)	13 (15.7)	0 (0)
Anastomosis location				
Upper rectum	166 (54.2)	121 (61.7)	35 (42.2)	10 (37)
Mid rectum	77 (25.2)	51 (26)	20 (24.1)	6 (22.2)
Low rectum	63 (20.6)	24 (12.2)	28 (33.7)	11 (40.7)
Anastomosis				
ICA	306 (100)	196 (100)	83 (100)	27 (100)
ECA	0 (0)	0 (0)	0 (0)	0 (0)
Converted to open or other MIS	0 (0)	0 (0)	0 (0)	0 (0)

All values are count (percentages), unless specified otherwise

*ICA* Intracorporeal anastomosis, *ECA* Extracorporeal anastomosis, *MIS* Minimally invasive surgery

The median time to return of bowel function was 20 h (range, 4–96) and the mean length of stay was 2.4 days (1–15). There overall postoperative complications rate was 12.4%. Eight patients (2.6%) experienced postoperative ileus. There were no superficial or deep surgical site infection (SSI). Eleven patients (3.6%) developed organ SSI space including 5 patients with intra-abdominal abscess and 4 patients with anastomotic leak. There was one mortality (0.3%) due to toxic megacolon from resistant Clostridium difficile. The 30-day reoperation rate was 2.9% (*n* = 9) including six patients presenting with organ space SSI and three patients for postoperative obstruction at diverting loop ileostomy site. There were no complains of fecal incontinence at the time of the last postoperative follow up in any patient. However, formal anatomical and functional pelvic floor analysis was not performed in this cohort. Postoperative outcomes are in Tables 5 and 6.

**Table 5:** Postoperative outcomes of patients undergoing robotic NICE procedure

	All cases *n* = 306 (100)	Diverticulitis *n* = 196 (64.1)	Neoplasm/cancer *n* = 83 (27.1)	Others *n* = 27 (8.8)
Return of bowel function in hours, median (range)	20 (4–96)	19 (5–96)	21.5 (4–60)	N/p
Length of Hospital Stay in days, mean (range)	2.4 (1–15)	2.5 (1–14)	2.0 (1–15)	N/p
Postoperative ICU	1 (0.3)	0 (0)	1 (1.2)	0 (0)
30-day readmissions	21 (6.9)	12 (6.1)	5 (6)	4 (14.8)
30-day reoperations	9 (2.9)	8 (4.1)	1 (1.2)	0 (0)
30-day mortality	1 (0.3)	1 (0.5)	0 (0)	0 (0)

N/p Not reported due to combined specialty surgery

All values are count (percentages), unless specified otherwise

*NICE* Natural orifice intracorporeal anastomosis with transrectal, *ICU* Intensive care unite, *SSI* Surgical site infection

**Table 6:** Postoperative complications

	All cases *n* = 306 (100)	Diverticulitis *n* = 196 (64.1)	Neoplasm/cancer *n* = 83 (27.1)	Others *n* = 27 (8.8)
Overall postoperative complications	38 (12.4)	27 (13)	7 (8.4)	4 (14.8)
Atrial fibrillation	1 (0.3)	1 (0.5)	0 (0)	0 (0)
Thrombosis	1 (0.3)	0	1 (1.2)	
Stroke	1 (0.3)	1 (0.5)	0 (0)	0 (0)
Blood transfusion	1 (0.3)	1 (0.5)	0 (0)	0 (0)
Dehydration/High output ileostomy	3 (1) *	3 (1.5) *	0 (0)	0 (0)
Urinary tract infection/pyelonephritis	2 (0.7)	2 (1)	0 (0)	0 (0)
Anal fissure	1 (0.3)	1 (0.5)	0 (0)	0 (0)
Crohn’s flare	1 (0.3)	1 (0.5)	0 (0)	0 (0)
C difficile colitis	3 (1)	3 (1.5)	0 (0)	0 (0)
Perianal abscess drainage	1 (0.3)	0 (0)	1 (1.2)	0 (0)
Postoperative ileus	8 (2.6)	6 (3)	2 (2.4)	0 (0)
SSI				
Superficial	0 (0)	0 (0)	0 (0)	0 (0)
Deep	0 (0)	0 (0)	0 (0)	0 (0)
Organ Space	11 (3.6)	6 (3)	1 (1.2)	4 (14.8)
Obstruction at diverting ileostomy site	3 (1)	2 (1)	1 (1.2)	0 (0)
Respiratory insufficiency	1 (0.3)	0 (0)	1 (1.2)	0 (0)

All values are count (percentages), unless specified otherwise

*SSI* Surgical site infection

*Percentage reported based on entire cohort. When calculated based on the subset of 40 ileostomy patients, there was a 7.5% rate of high ileostomy output dehydration

Table 7 depicts the subset of patients (*n* = 83) with colorectal neoplasm. The median tumor size was 3 cm (range, 1–9). The mesorectal grade was complete in all cases. Distal and proximal margins were negative in all cases. One patient had positive microscopic disease at circumferential margin (pT4 stage). This patient had known distant disease and had received neoadjuvant chemotherapy. The median examined lymph nodes were 19 (range, 6–36) and 37.3% patients had node positive disease. Recurrence and survival outcomes are not reported due to insufficient follow up interval.

**Table 7:** Subset of patients with neoplasm or cancer undergoing Robotic NICE procedure

	*n* = 83 (%)
Preoperative chemotherapy	7 (8.4)
Abdominal site extraction due to bulky specimen	13 (15.7)
Surgical pathology	
Adenoma	3
No lesion or no residual cancer	6
AJCC stage 0	3
AJCC stage 1	17
AJCC stage 2	15
AJCC stage 3	32
AJCC stage 4	7
Examined lymph nodes, median (range)	19 (6–36)
Presence of positive lymph nodes	31 (37.3)
Circumferential Resection Margins	
Compromised (≤ 1 mm) please see below	1 (1.2)
Uncompromised (> 1 mm)	82 (98.8)
Clear distal margins (> 2 cm)	83 (100)
Clear proximal margins (> 2 cm)	83 (100)
Tumor size in cm, median (range)	3.0 (0.7–9.0)

All values are count (percentages), unless specified otherwise

*NICE* Natural orifice intracorporeal anastomosis with transrectal extraction, *AJCC* American joint committee on cancer

## Discussion

Franklin et al. were the first to describe laparoscopic sigmoid resection with intracorporeal anastomosis and transrectal extraction of specimen, in 1993 [5]. Over the next two decades, several case series reporting feasibility and safety of laparoscopic natural orifice specimen extraction (NOSE) procedures were published [14–19]. In two randomized control trials, one including malignant and the other including both benign and malignant left-sided disease, the laparoscopic NOSE arm had less postoperative pain, lower use of opioids, lower rates of wound infections, and better cosmesis compared to the conventional laparoscopic arm [8, 10]. In spite of the enthusiasm with natural orifice techniques in left-sided colorectal resections, adaptation has been limited chiefly due to technical barriers and there has been a general consensus that these procedures should be reserved for selected patients only [20, 21].

In 2018 we published the first report describing the application of daVinci Xi® robotic technology for this natural orifice total intracorporeal surgery and termed it the NICE Procedure [11, 12]. We thereafter developed a stepwise technique with ten defined steps to facilitate reproducible results in an expanded patient population [13]. As such the NICE procedure has become our preferred approach regardless of patient characteristics such as age, ASA, BMI or disease type. In this report, we present the largest case series of robotic NICE procedure on consecutive and unselected cohort of patients undergoing left-sided colorectal resections for benign and malignant disease.

This was an intention to treat cohort and we therefore excluded patients in which we planned to perform a diverting loop ileostomy such as those who had undergone neoadjuvant radiation for rectal cancer. In these patients, we use the ileostomy site to extract the specimen. In our series however, we had a subset of 40 patients in which a decision was made to perform a diverting loop ileostomy intraoperatively. In most of these cases, the decision was made following the natural orifice extraction and based on the integrity of the anastomosis as well as clinical features of the patient. In this subset of 40 patients, all underwent successful ICA and all but 2 cases, in which bulky malignant disease was present, had transrectal extraction of the specimen. We feel that transrectal extraction in these cases still serves several advantages and avoids manipulation and extension of the incision at the ileostomy site.

Although BMI greater than 30–35 kg/m2 has been reported to be a limitation for successful transrectal extraction of specimen, [10, 18, 20–23] we have not found this to be the case. In our cohort of unselected patients 34.3% of the patients had BMI OVER 30 kg/m^2^ which is representative of a typical patient population in Texas as reported by the CDC. [24] Obesity was not associated with inability to extract the specimen or perform an ICA. In fact, the formation of an abdominal extraction incision is typically more cumbersome in those with large abdominal girth. Additionally, the extracorporeal portions of the anastomosis may be fraught with difficulty due to limitations of the tissue reaching above the abdominal wall. Since obesity is associated with increased risks of SSI as well as higher rates of incisional hernia formation, obese patients might benefit the most from avoidance of an abdominal wall incision [25–30].

It has been suggested that ICA and TRSE during robotic colorectal resection is associated with increased operative time [6]. However, these studies were performed on the Si daVinci® platform which has limitations resulting from instrument clashing and the propensity for re-docking to gain access to the left upper quadrant for splenic flexure mobilization. The Xi daVinci® platform was chiefly utilized during our study and we found the medium operative time of 219 min fared well when compared to published operative times ranging from 130 to 256 min for robotic colorectal left-sided resections with ECA and transabdominal extraction.[31–34, 34–40]. Furthermore, for benign disease, we perform mesenteric sparing dissection which is an efficient technique that avoids the need for exposure and access to the retroperitoneal structures [41]. Adhering to the stepwise process for completing the intracorporeal portions combined with the absence of creating and closing an abdominal wall extraction site results in the optimization of the operative time. Additionally, more recent advances in instrumentation such as the vessel sealer extend and upgrades in the energy generator yields faster division of the tissue, hemostasis, and other surgical maneuvers.

When assessing success rates of the NICE procedure we found that all consecutive patients were able to undergo an intracorporeal anastomosis. We predominantly used the 31 mm circular stapler because the larger chamber can receive additional tissue that usually results from a double purse-string closure of the proximal bowel and rectum. In 20 patients we opted for a handsewn end-to-end anastomosis to preserve the bowel length in the presence of possible tension in those with colorectal cancer (*n* = 3) or in cases in which numerous large-mouth diverticula were present at the anastomotic site (*n* = 17). All but one patient (99%) with benign pathology had successful TRSE compared to those presenting with malignant disease (84.3%). This high success rate is consistent with reports that identify the benign disease as a favorable indicator of achieving TRSE. In about 25% of those with benign disease, the specimen was bulky and required reduction prior to extraction by thinning of the mesentery. It is important to note that we do not thin or manipulate the specimen in any patient in which there is a suspicion of malignancy based on preoperative evaluation or intraoperative findings. In the one patient with bulky benign disease, we opted not to shave the mesentery because pathology was in question and therefore, we performed an abdominal extraction. Thirteen patients with malignant disease required abdominal wall extraction due to large cancer size and/or bulky mesentery. Thinning of the mesentery is not an option in this cohort. Although it has been reported that the upper limit of tumor size is from 4 to 6 cm [8, 9, 22, 42]. for TRSE, we found that size of tumor did not necessarily correlate with success rate because even small tumors may be associated with a very thick and bulky specimen and therefore not appropriate for natural orifice extraction.

Factors other than BMI, presence of benign disease, and small tumor size that have been associated with successful natural orifice transrectal extraction include female gender and the location of the disease in the distal colon or rectum [20, 48]. We found similar tendencies in our series. The success rates of TRSE was greater in females and among the 14 patients in which TRSE was not successful, 9 (64%) were male. In addition, all patients in our series had distal disease in the left colon, sigmoid and rectum which likely contributed to the overall high success rates of TRSE.

The median return of bowel function was 20 h, and the median LOS was 2.4 days. In published works reporting laparoscopic NOSE, the length of stay varied from a range of 1.2–3.5 days, and from 4.2 to 11.8 days [6–10, 42–44]. These reports are across various techniques, patient selection and recovery protocols which could explain the variation in results [10, 45]. We enter all patients into an multimodal opioid sparing, enhanced recovery protocol, what could contribute to our cohorts’ improved postoperative recovery. Only 2.6% patients experience post-operative ileus. Our overall complication rate was 12.4% including 3.6% with SSI. This compares favorably to SSI superficial and deep rates of 4.7–6.2% in conventional robotic ECA surgery [31, 37, 46]. There were no occurrences of superficial or deep SSI which is a direct reflection of avoiding an extraction incision.

In conclusion, the NICE procedure is associated with a very high success rate in regard to both intracorporeal anastomosis and transrectal specimen extraction in a large cohort of unselected and consecutive patients. This stepwise approach using the robotic platform may facilitate widespread adoption of this natural orifice total intracorporeal surgery with numerous inherent patient benefits.
